# Equal–Additive–Subtractive Remanufacturing Integrated Laser Directed Energy Deposition with Shot Peening and Machining Induced High Performance of Plunger Rod

**DOI:** 10.3390/ma17194767

**Published:** 2024-09-28

**Authors:** Xiaoyu Zhang, Wenping Mou, Dichen Li, Shaowei Zhu, Lianyu Li, Qiaochu Liu, Sheng Huang

**Affiliations:** 1 Chengdu Aircraft Industrial (Group) Co., Ltd., Chengdu 610092, China; 2 State Key Laboratory for Manufacturing System Engineering, Xi’an Jiaotong University, No. 28 Xianning West Road, Xi’an 710049, China; 3 School of Mechanical Engineering, Xi’an Jiaotong University, No. 28 Xianning West Road, Xi’an 710049, China

**Keywords:** equal–additive–subtractive technology, plunger rod, remanufacturing, residual stress, machining defects

## Abstract

The number of easily destroyed parts with high value is increasing in industry, and green remanufacture engineering is now mainstream in this new and expanding industrial field. Equal–additive–subtractive manufacturing, as a new technology that combines strengthening technology, additive manufacturing, and machining technology has great potential for development in the area of remanufacturing. Aiming at the damage characteristics of a plunger rod, this paper carries out a study about the repair technology by equal–additive–subtractive manufacturing of laser-directed energy deposition and shot peening. It was found that the microstructure of the materials repaired by equal–additive–subtractive technology is finer and the tensile strength can reach 100.4% of the base material. The surface residual stress of cladding materials changes from tensile stress to compressive stress, which reduces forming defects. Equal–additive–subtractive manufacturing has great significance in expanding the application of hybrid manufacturing and promoting green remanufacturing of parts with high value.

## 1. Introduction

With the rapid development of the economy and technology, the number of damaged parts with high value in industry has increased year by year, and the high amount of scrap parts has led to a huge loss of economic value. The transformation of remanufacturing for green development has become one of the mainstream trends in the manufacturing industry [1,2,3]. Additive manufacturing technology, as a rapidly rising advanced manufacturing technology in the past 30 years, can realize the manufacturing of complex entities by discretizing three-dimensional data and adding two-dimensional forming paths, which has important applications in aviation, aerospace, military, and other remanufacturing fields [4,5,6]. Laser-directed energy deposition has great potential in the field of metal repair. For the characteristics of joining and layered forming in additive manufacturing, it is difficult for the surface quality of forming parts to meet the precision requirements in use, and the forming parts generally need to be machined. Fox et al. [7] formed stainless steel by laser powder bed fusion, and the measured surface roughness exceeded Ra30, making it difficult to meet the requirements in use. Afazov et al. [8] formed Inconel 718 parts by laser powder bed fusion and reduced distortion from approximately ±300 μm to approximately ±65 μm for both components, which still cannot be used directly. Laser additive manufacturing is a kind of thermal forming process and the residual tensile stress occurs during the rapid melting and solidification of materials. In addition, it is difficult to avoid the appearance of micro-defects or cracks in the forming process, which leads to processing defects in the subsequent machining process, affecting the use and delivery of parts. Mumtaz et al. [9] produced high-density Waspaloy^®^ specimens by selective laser melting, which were 99.7% dense. Vilaro et al. [10] formed TC4 by laser powder bed fusion and found defects inside the material by microscopic observation. Due to the above reasons, the application of additive manufacturing is limited.

Researchers carried out studies on the defects and shortcomings of additive manufacturing in order to improve its usability in applications. At first, researchers expected to improve the forming characteristics through process optimization, but they could not solve the problems of overall joint effect, thermal stress, and micro-defects that occurred in additive manufacturing [9,10]. Then, researchers tried to solve the problems from the manufacturing process itself, proposing a new concept of equal–additive–subtractive manufacturing, which combined subtractive manufacturing, surface-strengthening technology, and additive manufacturing to achieve high-precision and high-quality manufacturing capabilities. Based on additive–subtractive manufacturing, the method integrates surface strengthening techniques called equal manufacturing, such as micro-forging, laser shock, shot peening, etc. Zhang et al. proposed a similar concept also known as “micro-casting forging and milling” [11]. The advantages of this method are removing the surface stress and microdefects of the materials in additive manufacturing by strengthening layer by layer and improving the accuracy by machining. The concept of hybrid additive manufacturing was first proposed by Prinz in a patent, in which it described a method to form suspended structures with support features by spraying, and guaranteed accuracy by hybrid manufacturing with machining [12]. Akula et al. [13] and Karunakaran et al. [14] combined arc additive manufacturing technology with numerical control machining technology to improve the accuracy of parts through the cycle of forming and machining, and tested the performance of formed samples, which still had a gap compared with that processed by traditional manufacturing. Kerschbaumer and Ernst [15] integrated the laser cladding nozzle and powder feeding system into the CNC machine tool to reduce the flow phenomenon during the forming process with the flexibility of the five-axis movement and achieve high-precision manufacturing by alternating milling. However, the cutting fluid could not be used during the manufacturing process due to the influence of raw materials, which reduced the manufacturing efficiency. In terms of strengthening performance in hybrid manufacturing, B.N. Mordyuk et al. [16] combined high-frequency ultrasonic impact with laser-directed energy deposition and found that the coarse dendrite generated in the forming process could be broken and refined by high-frequency ultrasonic impact, and the tensile stress was transformed into compressive stress at the same time. However, the effect of ultrasonic impact was affected by the forming surface. In the actual manufacturing process, the surface of the forming layer is not uniform and the wear of the impact head could seriously affect the impact load. Kalentics et al. [17] combined laser selective melting with laser shock strengthening, formed 316L samples, and tested the residual stress, finding that the introduction of high compressive stress at some depth was conducive to improving material properties. However, the process could only be completed with the aid of water film or a large number of shocks, which reduced the manufacturing efficiency. Zhang et al. [18] proposed a new green manufacturing method by combining arc additive manufacturing, rolling strengthening, and milling technology to strengthen the material in the thermoplastic area of the deposited layer to eliminate internal holes and stress deformation, and ensure manufacturing accuracy through milling. However, the grain size in the surface area was relatively coarse. The strengthening effect of the sidewall was limited. At present, there are still some limitations in the practical application of various hybrid manufacturing technologies.

To solve these problems, we propose a hybrid remanufacturing technology based on laser-directed energy deposition, shot peening, and milling. On the one hand, the problem of insufficient strengthening effect in hybrid manufacturing is avoided by the flexible method of shot peening, and on the other hand, the process interference in the additive and subtractive manufacturing process is avoided by the method of serial manufacturing. A comparative study on the microstructure and properties of the repaired plunger rod for a high-pressure oil pump is carried out, and the damaged plunger rod is repaired by a hybrid process. The repair process based on equal–additive–subtractive technology is of great significance for expanding the application range of additive manufacturing, and can effectively promote the development of the green remanufacturing industry.

## 2. Materials and Method

### 2.1. Materials

The experiments and repair verification are carried out for the plunger rod of a high-pressure oil pump in this paper. The main function of the plunger rod is to transport fluid to the high-pressure oil pump. The front of the plunger rod is threaded to the cylinder block. There is a sealing element at the fit clearance of the plunger rod and the cylinder block to prevent fluid from overflowing. When working, the plunger rod pulls back, then the pipe valve at the outlet is closed, the pipe valve at the inlet is open, and the fluid enters the cylinder due to the suction. When the plunger rod is pushed forward, the fluid in the cylinder is compressed out. By the reciprocating movement of the plunger rod, the fluid can be continuously transported to the target.

**Figure 1:**
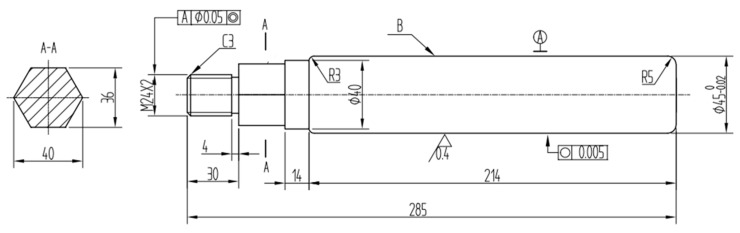
The dimensions of the plunger rod.

The plunger rod is manufactured by turning. Its heat treatment processes are hardening and tempering. The hard coatings are deposited on side B with hardness of more than HRC 55. The dimensions of the plunger rod are shown in Figure 1. The dimensional tolerances not indicated in the figure are −0.2 mm to +0.1 mm. The surface roughness not marked in the figure is Ra3.2.

**Figure 2:**
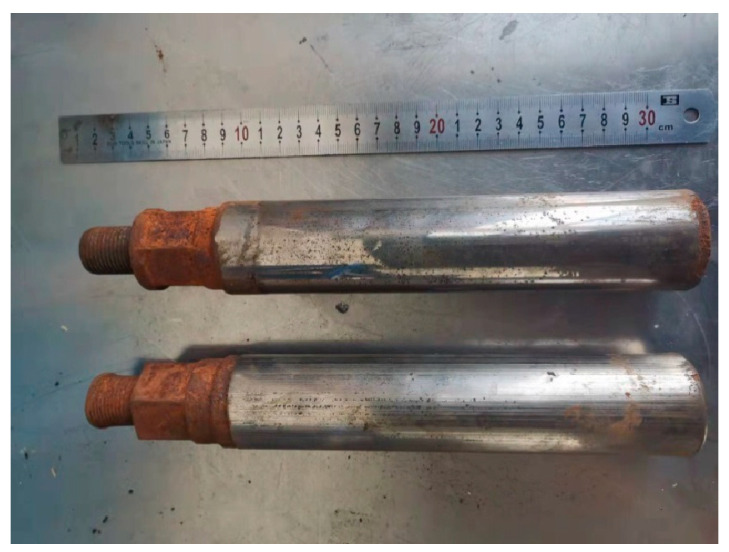
Damaged plunger rod.

The damage to the plunger rod mainly includes surface corrosion, surface wear, and compression deformation. The damaged plunger rod is shown in Figure 2. The plunger rod has contact with the corrosive fluid when working, the front of which is used as a matching mechanism to contact the fluid in the cylinder. With the joint action of corrosive fluid and air, the surface has a more serious corrosion phenomenon, which is the main reason for the failure of the plunger rod. In addition, the front is affected by high pressure, the plunger rod deforms easily, and the thread of the plunger rod is shown in the figure, which has obvious deformation. Due to the reciprocating movement of the plunger rod, the surface wear of the shaft damages easily. The wear is reduced by depositing wear-resistant coatings with chromium, and generally, the service life of the shaft is higher than that of the front. The plunger rod must be stopped for inspection within a period of use, affecting normal operation.

The material of the plunger rod is 40 Cr steel(forging). The material of the plunger rod is under quenching and tempering treatment, and the composition is the same as that in the published paper [19]. 40 Cr(forging) is a medium carbon steel with modulation, mostly used for the material of shafts, which has good machinability. The material has a good strength and toughness after processing. The tensile strength is shown in Table 1. 40 Cr(forging) has weldability, on which the repair materials can be deposited.

For the selection of repair materials, the repair method of the same material is generally preferred. However, carbon steel is more sensitive to the cooling rate, and a large number of brittle and hard martensitic microstructures occur in the process of laser-directed energy deposition. It is difficult to achieve microstructure control through adjustment of process parameters, and heat treatment is required to toughen it. In addition, carbon steel is more sensitive to oxygen, and oxidation occurs easily in an open environment or field repair. The repair material of the plunger rod is Fe314, whose composition is shown in Table 2. The material has a good match for most medium and low carbon steel, and has a good metallurgical bond at the interface of the weak position of the repair, without cracking. The B in the material has a good deoxidation effect, which avoids the oxidation phenomenon in the repair and forming process. The same material is used for shot peening to avoid impurities during forming.

The hybrid process studied in this paper can provide technical support for the development of metal additive manufacturing technology and can be applied to other material systems. The material used in this paper is stainless steel, which has a softening phenomenon at a temperature, which can achieve better strengthening effects by using the hot shot peening method of the same material. For other materials with thermoplastic temperatures, such as tantalum alloy, the hybrid process can also be used by the method of argon shielding and carrying powders. For tungsten alloy and other hard metals, the thermoplastic characteristics are not obvious, and the strengthening effect of the hybrid process is limited.

### 2.2. Experimental Scheme and Equipment

**Figure 3:**
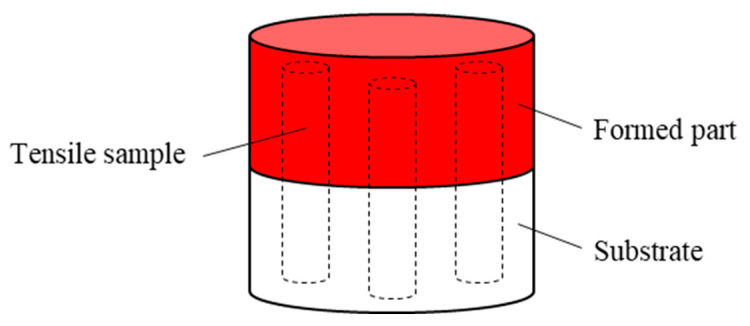
The forming scheme.

**Figure 4:**
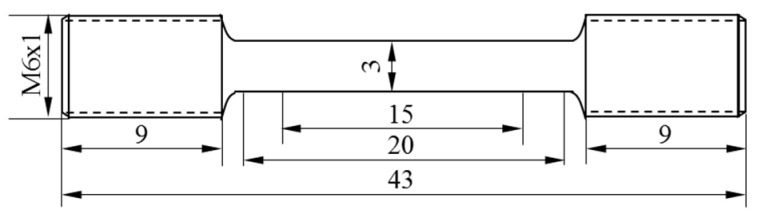
Size of the tensile sample.

To compare and evaluate the difference between laser-directed energy deposition and the hybrid process in the repair effect of the plunger rod, Ф30 × 25 mm cylinders are formed by two processes on 40 Cr(forging) cylinders with a height of 25 mm, and three tensile samples are taken from each cylinder with half a forming part and half substrate. The repair interface is located in the middle of the tensile sample. The forming scheme is shown in Figure 3. The forming equipment is self-made hybrid equipment of laser-directed energy deposition and shot peening. The formed samples are machined into tensile samples for testing. The size of the tensile samples is shown in Figure 4. The tensile test standard is GB/T 228.1-2020 [20]. The sample with a size of 15 × 15 × 2.5 mm is formed on a 40 Cr(forging) substrate with a thickness of 10 mm, and the density of the sample and the substrate is tested to evaluate the effect of the hybrid process on the defects at the bonding interface. The density is measured by the drainage method. The section perpendicular to the deposition direction is ground, polished, and etched, and the etching solution is HCl:HNO_3_:H_2_O = 1:1:1. The microstructure is observed and analyzed by Keyence VH-600 optical microscope(KEYENCE, Osaka, Japan), and the bonding interface is observed. HXD-2000TM/LMD micro-hardness tester(Shanghai Taiming Optical instrument Company, Shanghai, China) is used to test the hardness of the section. The load is 500 g, the load retention time is 10 s, the test times are 3, and the test interval is 0.2 mm.

**Figure 5:**
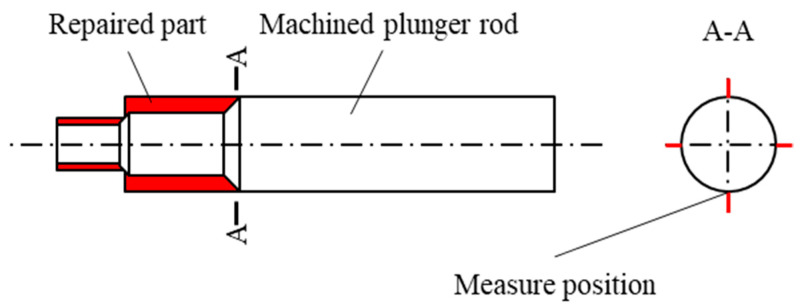
The repair and inspection scheme of the plunger rod.

The plunger rod repair and testing scheme is shown in Figure 5. The front of the plunger rod is cut to remove the rust surface and deformation, and the repair is carried out by rotating the additive manufacturing method. After the repair, the machining is carried out to ensure the dimensional accuracy. The surface stress of the repaired plunger rod is measured. As the bonding position of different materials, the stress concentration occurs near the bonding interface, which affects the forming properties. The surface stress near the repaired interface is detected by the method of X-ray stress measurement by X-350A equipment (Aisite Institute, Handan, China). The residual stress around the interface is measured at the quartered position, the stress direction is measured along the axial direction, and the average stress is calculated. The stress constant is −601 MPa, the scanning angle 2θ is 134.00°~124.00°, the tube voltage is 25.0 kV, and the tube current is 5.0 mA. We also used the same detection methods and parameters in our previous study [21]. The detection position is shown in Figure 5. At the same time, the surface roughness of the corresponding position is detected. The roughness detection equipment is the TR300 roughness measuring instrument(Beijing Shidaifeng Technology Co., Ltd., Beijing, China), and the roughness detection direction is along the axis.

After repair, the plunger rod is machined to the required dimensions. On the one hand, the front thread of the plunger rod cannot be formed by laser-directed energy deposition. On the other hand, the front of the plunger rod is a high-precision mating surface, which the hybrid manufacturing cannot achieve. Therefore, the repaired plunger rod needs to be machined to meet the assembly accuracy requirements, to avoid the rapid failure of parts or damage to the plunger pump due to poor assembly accuracy in its use, and to improve the applicability of repaired parts.

### 2.3. Process Parameters

The forming process parameters of the samples are studied and optimized [22]. In the hybrid process, the pressure of shot peening is 4 bar, the injection angle is 45°, and the hybrid forming method of shot peening for each layer of deposition is adopted. The process parameters of the experiment are shown in Table 3.

In the actual process of plunger rod repair, for the way of rotary repair, the motion parameters optimized under the plane need to be transformed into rotary motion parameters. After pretreatment, the diameter of the step shaft is 35 mm and 20 mm, and the length is 41 mm and 30 mm, respectively. The rotary process parameters after conversion according to Table 3 are shown in Table 4, in which the laser power, laser spot size, and powder feeding rate are all adopted in Table 3. The forming time of this process in Table 3 is about 72 min. The machining time of the part after deposition is about 120 min. The total repair time is about 192 min. The manufacturing time of a new plunger rod is approximately 527 min including machining, depositing hard coatings, and heat treatment. With this remanufacturing method, the efficiency can be increased by 64%. When the height of the repaired deposition reaches 28 mm, the repair time of the plunger rod is roughly the same as the manufacturing time of the new part; that is, when the depth of the surface defects of the plunger rod reaches 28 mm, the method of manufacturing the new part is more efficient.

## 3. Results

### 3.1. Microstructure and Mechanical Properties

#### 3.1.1. Microstructure

**Figure 6:**
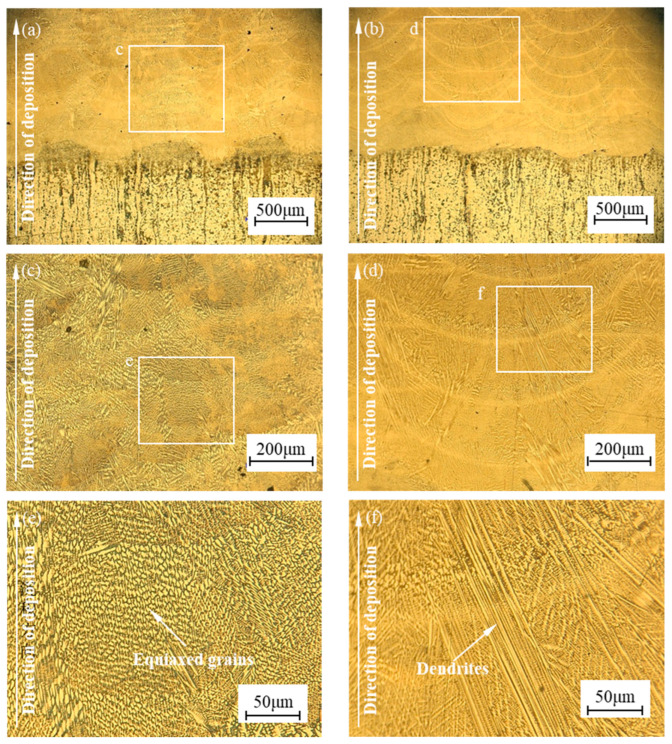
Comparison of the microstructure of repaired samples: (**a**) The sample repaired by hybrid process, (**b**) The sample repaired by single process, (**c**) Multilayer diagram of sample repaired by hybrid process, (**d**) Multilayer diagram of sample repaired by single process, (**e**) Internal diagram of sample repaired by hybrid process, (**f**) Internal diagram of sample repaired by single process.

The bonding interface and microstructure of the samples repaired by the two processes are observed through an optical microscope, and the comparison of the microstructure of repaired samples is shown in Figure 6. The bonding interface is one of the most important positions of the repairing part, and poor bonding of the repairing interface can easily lead to the failure of the part due to the interface cracking during use. As shown in Figure 6a,b, both samples bond well with the substrate, which is metallurgical bonding without obvious cracking phenomenon. Affected by the linear cutting marks on the surface of the substrate, the shape of the bonding interface is wavy. Regular deposited paths can be observed in samples formed by the single process, and the arrangement is relatively regular. Due to plastic deformation, the deposited paths in the sample formed by the hybrid process are more compact, and the width and height of the paths are changed.

As shown in Figure 6c–f, the microstructure of the samples is mainly an eutectic structure, and a large number of dendrites can be observed in the sample formed by laser-directed energy deposition. Due to the influence of heat accumulation, some dendrites grow continuously from inside the cladding layer and pass through the joining area. The number of dendrites decreased in the sample formed by the hybrid process. Due to the plastic deformation caused by shot peening and recrystallization caused by laser remelting, there are a large number of equiaxed crystals in the sample formed by the hybrid process, which makes the microstructure more fine. The results are the same as EBSD maps in a previous study [21]. There is no obvious change in the structure of the substrate. The thickness of the strengthening layer of shot peening is about 0.3–0.8 mm. During the first few layers of forming, only the surface of the substrate is strengthened. As the height of the deposited cladding layers increases, the substrate is no longer strengthened. Therefore, the structure of the substrate changes near the interface, and most of the structure does not change.

#### 3.1.2. Tensile Properties

**Figure 7:**
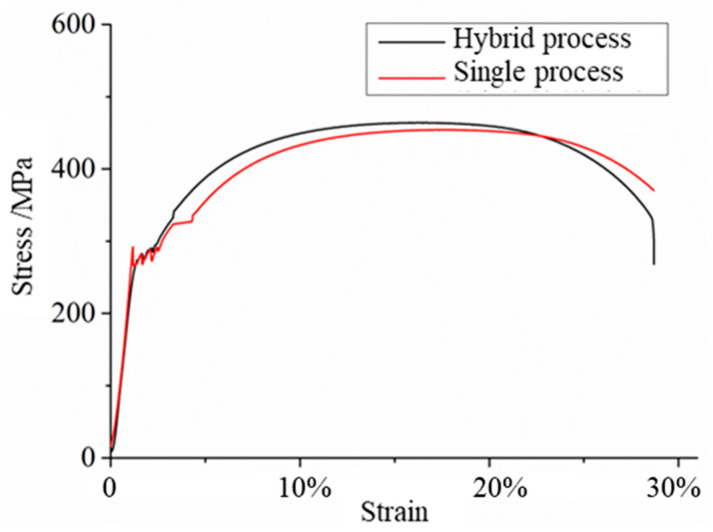
Tensile stress–strain diagrams of samples repaired by different processes.

To evaluate the mechanical properties of the repaired materials, tensile tests are carried out on the repaired samples. Table 5 shows the tensile properties of repaired samples. Figure 7 shows the tensile stress–strain diagrams of samples repaired by different processes. As shown in the figure, the stress fluctuates after entering the plastic regime and there is no obvious yield point. When the stress increases to the maximum, it decreases sharply until the final fracture occurs at the position of necking.

The fracture position of the repaired samples in both processes occurs at the base material, and no fracture occurs at the repaired interface position, indicating that the performance of the repaired interface is higher than that of the base material, and can meet the requirements of the use of the base material. Since the fracture occurs at the base material, the tensile curve of the sample is consistent with the properties of the base material, with good plasticity and moderate strength, and there is an obvious yield stage. After the stress increases to the maximum, the necking instability occurs, and then a fracture occurs. The properties of the samples repaired by the hybrid process reach 100.4% of the properties of the substrate, and the samples repaired by the single process reach 98.3% of the properties of the substrate, both of which reach more than 98% of the properties of the substrate, meeting the repair requirements. The tensile properties of the samples repaired by the hybrid process are close to those of the samples repaired by the single process, because the properties of the repaired materials are higher than those of the substrate, and the fractures occur at the substrate during the tensile process, thus showing the properties of the substrate. However, the hybrid process only has a strengthening effect on the repaired part, and the effect on the substrate is only surface strengthening, and the enhancement effect is limited.

#### 3.1.3. Hardness

**Figure 8:**
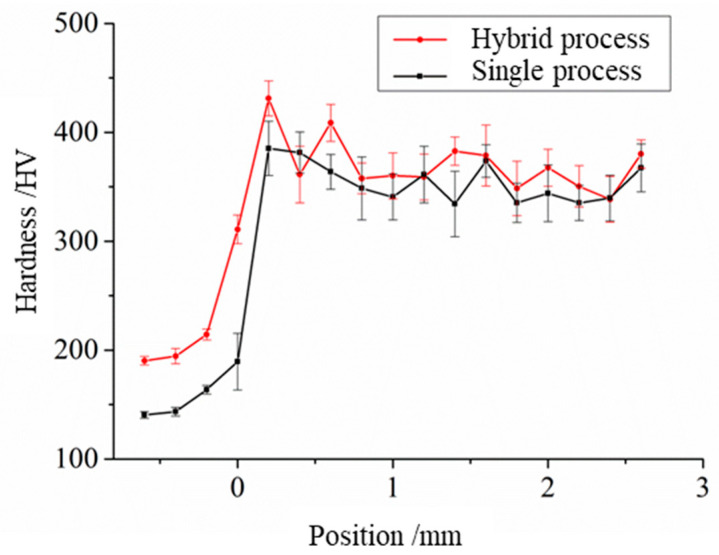
Hardness change in the repaired samples.

The samples repaired by two processes have differences in the hardness, which has an impact on the wear resistance of the repaired materials. Table 6 shows the hardness of the repaired samples. Figure 8 shows the hardness change in the repaired samples. The average hardness of the sample repaired by the hybrid process is higher than that of the sample repaired by the single process, which is increased by 4.7% because the hardness of the material is increased by shot peening in the hybrid process.

The hardness of the substrate surface in the hybrid process increases due to the impact of shot peening on the substrate. The hardness of the repaired part fluctuates, and the hardness changes in the inner and joining of the cladding layer show a soft and hard alternating law. In addition, the hardness strengthening phenomenon occurs at the interface due to component diffusion. The hardness increase in the repaired material under the hybrid process can effectively improve the wear resistance of the parts, especially for the plunger rod, improving its service life effectively.

#### 3.1.4. Density

The density of the material can indicate the internal defects. The density of the samples repaired by the two processes is tested, and the density of the samples is shown in Table 7. The density of the sample repaired by the hybrid process is 99.8% of the density of the substrate, which is slightly higher than that by the single process. On the one hand, the volume of substrate accounts for two-thirds of the sample, and the substrate forges with few internal defects, making the density of the sample close to that of 40 Cr(forging). The hybrid process can mainly decrease the number and size of the internal defects of the formed samples, and thus has an effect on improving the density of the samples. Because the repaired part only accounts for one-third of the volume of the sample, the density of the two samples has a small difference, and the improved effect is not obvious. On the other hand, the repaired material and 40 Cr(forging) have good metallurgical bonding, and the interface of the sample repaired by laser-directed energy deposition is good, almost with no defects, and it is difficult to achieve a higher density enhancement effect in the hybrid process. However, the density test results are also consistent with the observed results at the interface, and the repair quality of the two processes is good, and there are no obvious defects at the interface.

### 3.2. Repair of Plunger Rod

#### 3.2.1. Macroscopic Appearance

**Figure 9:**
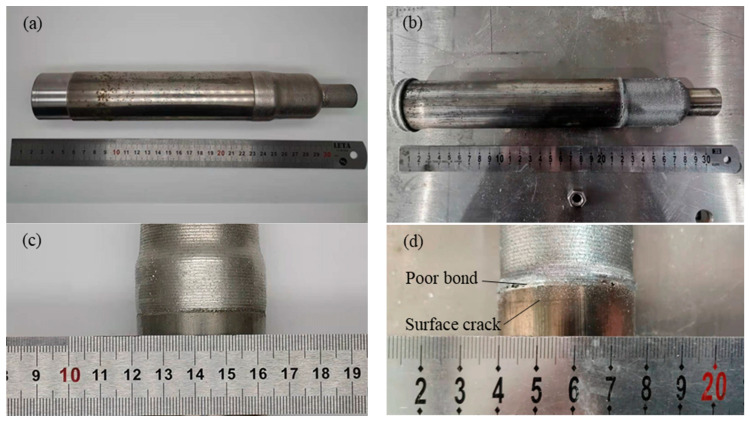
The initially repaired plunger rod: (**a**) Plunger rod initially repaired by hybrid process, (**b**) Plunger rod initially repaired by single process, (**c**) Partial view of the plunger rod initially repaired by the hybrid process, (**d**) Partial view of the plunger rod initially repaired by the single process.

The macroscopic appearance of the plunger rod after initial repair by the two processes is observed. The plunger rod after initial repair by laser-directed energy deposition and the hybrid process is shown in Figure 9, in which Figure 9a,c show the plunger rod after initial repair by the hybrid process, and Figure 9b,d show the plunger rod after initial repair by the single process. As shown in the figure, the surface of the initially repaired part of the plunger rod shows a bright metallic color, and no obvious oxidation occurs during the repair process. The joining paths on the surface of the initially repaired plunger rod are weakened by abrasion of shot peening in the hybrid process. Moreover, due to the abrasive effect of shot peening, the transition position of the step shaft of the plunger rod is smoother.

Since the interface is the weak position of initial repair parts, the partial view of the bonding interface is observed in Figure 9c,d. It can be seen from the figure that the interface initially repaired by the hybrid process has good bonding without obvious defects. At the initial repair interface in the laser-directed energy deposition, the phenomenon of a poor bonding interface can be obviously observed, and cracks are observed on the surface of the unrepaired part of the plunger rod near the initial repair interface. The reciprocating movement of the plunger rod easily wears the surface of the shaft, and coatings with Cr are deposited on the surface of the plunger rod to increase the service life. The material of coatings is a medium or high carbon steel with strong wear resistance, which is hard and brittle. The coatings of the shaft may be affected by the thermal stress at the interface and crack occurs on the surface. In addition, the materials of coatings have poor metallurgical bonding, which is also affected by thermal stress and poor bonding occurs at the interface.

#### 3.2.2. Residual Stress

The residual stress on the surface of the plunger rod repaired by the two processes is detected, and the results of the residual stress on the repaired interface of the plunger rod are shown in Table 8. As shown in the table, large residual tensile stress occurs near the interface of the plunger rod repaired by the laser-directed energy deposition. The average compressive stress introduced by the hybrid process is about 1.8 times that of the tensile stress of the sample repaired by laser-directed energy deposition. The hybrid process has a good effect on improving the surface stress of the repaired parts. For shafts, heat transfer can only be carried out by convection through the fixture and the air. The plates can transfer heat to the working platform through the contact surface, and generally, the shafts easily produce a large heat accumulation when repaired compared with the plates, so the residual tensile stress of the material also increases.

The macroscopic appearance of the repaired plunger rod is related to the residual stress generated in the forming process. There is large residual tensile stress near the interface of the plunger rod repaired by the laser-directed energy deposition, and there are cracks on the surface and poor bonding at the interface, indicating that the cracks are due to the tensile stress generated on the surface of the plunger rod during the process of laser-directed energy deposition. The average axial residual tensile stress of the surface of the plunger rod is 423.1 MPa, which is close to 462 MPa, the average tensile strength of 40 Cr(forging). In the process of repair, the large residual tensile stress causes the cracking of the surface of the base material. Micro-cracks can easily cause the plunger rod to fail again in use.

The residual tensile stress at the chamfer of the interface of the different materials also causes poor bonding. This phenomenon does not appear in the microstructure observation sample, one is because the forming volume of the microstructure observation sample is small, and a lot of heat accumulation and residual tensile stress has not been generated. Secondly, the hard coatings after surface modulation treatment of the plunger rod have poor metallurgical bonding with the repair material, and the two materials have differences in plasticity, so the phenomenon of cracking and the appearance of defects at the chamber when the stress is concentrated occur easily.

In the hybrid process, due to the effect of shot peening, compressive stress appears at the surface of the plunger rod. At the same time, a large amount of cold air is introduced in the hybrid process, which weakens the heat accumulation and may also reduce the tensile stress generated in the forming process. Under the state of the surface compressive stress of the plunger rod, there is no cracking phenomenon on the surface, and the bad bonding phenomenon is eliminated.

**Figure 10:**
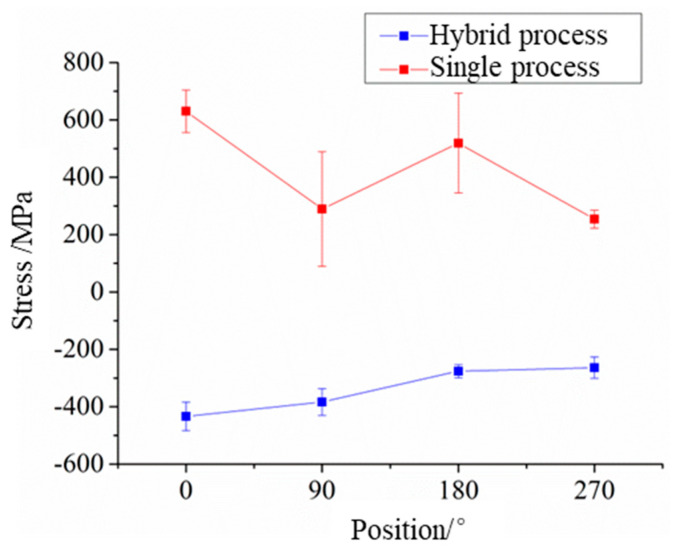
The distribution of surface residual stress near the interface of the repaired plunger rod.

The distribution of residual stress on the surface of the repaired plunger rod is studied. Figure 10 shows the distribution of surface residual stress near the interface of the repaired plunger rod. As shown in the figure, all the positions near the interface of the plunger rod repaired by the laser-directed energy deposition show tensile stress, and the tensile stress is the largest at the initial position. With the increase in the rotating angle of the plunger rod, the surface stress shows fluctuations. This is because, in the process of repairing the plunger rod, the laser always starts from the same initial position. In the process of starting the shaft rotation, there is an acceleration, so that the initial speed cannot reach the preset speed, and the large heat accumulation occurs at the initial position, resulting in a large residual tensile stress. According to the repair process, the starting range of rotation is about 0~3°. When the velocity is stable, the residual tensile stress is relatively stable and shows a fluctuation phenomenon.

In the hybrid process, the surface stress near the interface of the plunger rod is all compressive stress, and the compressive stress is the largest at the initial position. With the increase in the rotating angle, the compressive stress gradually decreases and tends to be stable. This phenomenon is also related to fixed initial points. In the process of shot peening, when the gas supply is stopped, part of the unejected powders is deposited in the shot peening pipeline. At the same time, the pressure vessel begins to recharge the gas to restore pressure. At the beginning of the shot peening, there is large gas pressure in the pipeline, and the powders deposited in the pipeline will be ejected together, introducing the initial position to stronger compressive stress. Therefore, there is large compressive stress at the initial position. As the peening process continues, the output of powders and pressure becomes stable, and the surface stress is stable too.

Compared with laser-directed energy deposition, the surface residual stress generated in the hybrid process is more stable, the standard deviation is smaller, and the fluctuation is relatively small. This is because the material has a limit of plastic deformation at one strength. The plastic deformation is generally largest at first when shot peening, and the strengthening effect will gradually weaken with the increase in the time of shot peening. In the hybrid process, the strength and effect of shot peening are relatively stable with a pressure of 4 bar. Even if there is a strong impact at the initial position, the fluctuation of surface stress is relatively small. However, in order to eliminate the stress concentration, this phenomenon can be weakened by changing the initial position in the subsequent research.

#### 3.2.3. Roughness

The surface roughness of the repaired plunger rod is shown in Table 9. The average surface roughness of the repaired plunger rod is Ra3.2 after the machining process in hybrid manufacturing, which can meet the requirements of the plunger rod. The average surface roughness of the plunger rod repaired by laser-directed energy deposition is Ra37.69. The problem of poor surface quality caused by joining and sintering powder on the surface in the single forming process can be effectively solved by machining technology. Hybrid manufacturing technology can effectively make up for the lack of precision of additive manufacturing and improve the quality of repair.

The residual stress and surface roughness of the plunger rod are both improved in the hybrid process. The beneficial surface roughness and compressive residual stress are considered the main factors that contributed to the improvement in the fatigue properties found in another study of surface strengthening [23]. This indicates that the hybrid technology proposed in this paper is expected to improve the fatigue performance of repaired parts. Although there is a bond line between the main material and the additive one, the fracture often occurs at the main material because the strength of the interface and the repair material is higher than that of the base material, which may reduce the possibility of fatigue fracture due to the delamination.

#### 3.2.4. Machining Quality

**Figure 11:**
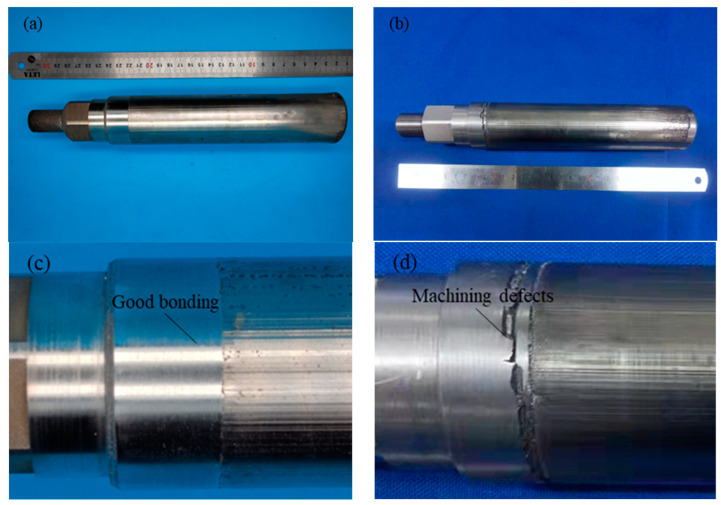
The machined plunger rods: (**a**) The machined plunger rods repaired by hybrid process, (**b**) The machined plunger rods repaired by single process, (**c**) Partial view of the machined plunger rods repaired by hybrid process, (**d**) Partial view of the machined plunger rods repaired by single process.

The machining process is mainly affected by the stress and defects generated in the repair process, and the machining defects may occur in the machining process. The machining allowance of the repaired plunger rod is 1 mm, and the surface machining roughness requirement of the plunger rod is Ra3.2. Due to the small machining allowance, the machining process of the plunger rod directly adopts the method of finishing, and the repaired plunger rods repaired by two processes are machined in the same way. The machined plunger rods are shown in Figure 11, where Figure 11c,d show the enlarged picture of the plunger rod near the repaired interface.

The surface of the plunger rod repaired by laser-directed energy deposition shows defects after machining. Due to the large tensile stress on the surface of the plunger rod repaired by laser-directed energy deposition, poor bonding occurs near the interface. In the machining process, the forming defects generated are affected by the tensile stress during the machining process, and the machining part of the surface of the forming material is peeled off due to the low bonding strength with the non-machining part, resulting in repair defects and making the repaired plunger rod unable to be put into use. The dimensional accuracy of the part does not meet the requirements due to the defects. The parts need to be repaired near the interface and then machined again to meet the size requirements.

There are no obvious machining defects on the surface of the plunger rod repaired by the hybrid process, and the size accuracy, position accuracy, and surface roughness of the part meet the requirements after machining. Due to the influence of the residual compressive stress on the surface, there are no repair defects between the deposited layer and the matrix. In the machining process, the non-machining part of the workpiece surface will not flake off due to tension, which can meet the processing and assembly needs of the plunger rod. The repaired plunger rod can meet the assembly requirements and run normally for a short time. After a comprehensive evaluation of the performance of the parts, a formal test will be carried out.

**Figure 12:**
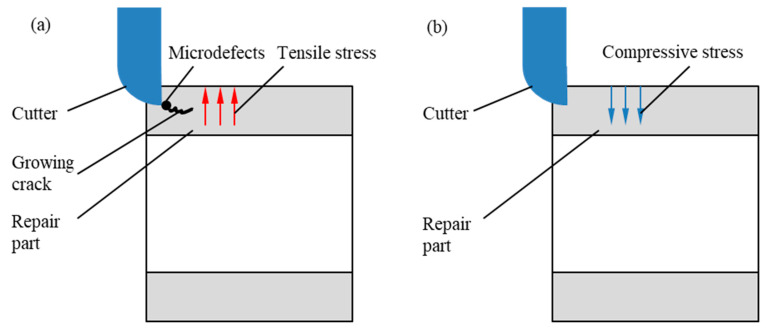
The machining process diagram of samples: (**a**) The machining process diagram of samples repaired by single process, (**b**) The machining process diagram of samples repaired by the hybrid process.

The microdefects and residual tensile stress have a great influence on the machining process. Figure 12 shows the machining process diagram of samples repaired by the two processes. In the machining process, when the tooltip moves near the position of the forming defect, under the joint action of the tool and the residual tensile stress, the internal defect of the material extends inward to form a micro-crack, which makes the materials crack along the direction of expansion in the processing process. And the internal non-machining part is peeled off, resulting in machining defects.

For the hybrid process, the density of the material is increased with few internal defects, and the surface stress of the material is compressive stress, which inhibits the generation of micro-cracks in the machining process. And it can effectively avoid the machining defects of the material. On the other hand, because the surface of the material in the hybrid process is affected by work hardening, the hardness of materials is increased. And the tool and processing parameters should be reasonably selected in the machining process to prevent the phenomenon of knife breaking and surface scratches.

## 4. Conclusions

In the present study, the effects of equal–additive–subtractive remanufacturing of laser-directed energy deposition and shot peening on the microstructure and properties of one repaired plunger rod are studied. Based on the experimental results and reasonable analysis, the following conclusions are drawn:

(1) The equal–additive–subtractive remanufacturing of laser-directed energy deposition and shot peening can effectively improve the microstructure of repaired materials, refine grains by introducing plastic deformation, and reduce coarse dendrites.
(3) The equal–additive–subtractive remanufacturing of laser-directed energy deposition and shot peening can improve the mechanical properties and hardness of the materials, and limited by the properties of the substrate, the tensile properties and density of the samples repaired by the hybrid process are slightly improved.
(3) The surface state of the plunger rod is improved by the equal–additive–subtractive remanufacturing of laser-directed energy deposition and shot peening, and the residual stress changes from tensile stress to compressive stress, and the surface roughness of the machined part, can meet the requirements.
(4) The residual stress on the surface of the plunger rod repaired by the equal–additive–subtractive remanufacturing of laser-directed energy deposition and shot peening is compressive stress. The bonding between the deposited layer and the matrix is dense and there is no crack on the substrate, which can effectively reduce the machining defects.

The method of equal–additive–subtractive remanufacturing proposed in this paper has a good effect on the repair of the damaged parts. In the future, it is expected to be used in the repair of more materials and parts with high value, expanding the application of hybrid manufacturing.

## Figures and Tables

**Table 1:** Tensile properties of 40 Cr(forging).

Material	Ultimate Tensile Strength/MPa	Yield Strength/MPa	Elongation/%
40 Cr(forging)	462 ± 2	267 ± 5	39.2 ± 1.0

**Table 2:** Components of Fe314 powders.

Element	Cr	Ni	B	Si	Fe
Mass fraction/%	15	10	1	1	Bal.

**Table 3:** Process parameters of the experiment.

Groups	Laser Power/W	Laser Spot Size/mm	Velocity/mm·s^−1^	Acceleration/mm·s^−2^	Joining Distance/mm	Layer Height/mm	Powder Feeding Rate/g·min^−1^	Height/mm	Shot Peening
1	1054	3	40	800	1	0.2	15.94	25	with
2	1054	3	40	800	1	0.2	15.94	25	without

**Table 4:** Rotary process parameters.

Groups	X-Velocity/mm·s^−1^	Rotate Speed/r·s^−1^	Angular Acceleration/r·s^−2^	Length/mm	Layer Height/mm	Height/mm	Shot Peening
1	0.38	0.36	7.2	41	0.2	6	with
2	0.38	0.36	7.2	41	0.2	6	without
3	0.69	0.64	12.8	30	0.2	5	with
4	0.69	0.64	12.8	30	0.2	5	without

**Table 5:** Tensile properties of repaired samples.

Process	Tensile Strength/MPa	Yield Strength/MPa	Elongation/%
Hybrid process	464 ± 7	288 ± 4	27.0 ± 0.5
Single process	454 ± 3	273 ± 6	28.5 ± 0.4

**Table 6:** Hardness of repaired samples.

Process	Average/HV	Maximum/HV	Minimum/HV
Hybrid process	371.2 ± 24.6	431.3	338.6
Single process	354.7 ± 17.0	385.3	334.3

**Table 7:** Density of samples.

Group	Hybrid Process	Single Process	40 Cr(forging)
Density/g·cm^−3^	7.834 ± 0.009	7.801 ± 0.014	7.85

**Table 8:** The residual stress of the repaired interface.

Process	Hybrid Process	Single Process
Residual stress/MPa	−339.6 ± 71.8	423.1 ± 156.9

**Table 9:** The surface roughness of the repaired plunger rod.

Process	Hybrid Process (Machined)	Single Process
Average of roughness Ra/μm	3.2 ± 0.01	37.69 ± 2.37

## Data Availability

The original contributions presented in the study are included in the article, further inquiries can be directed to the corresponding authors.
